# Strengthening integrated primary health care in Sofala, Mozambique

**DOI:** 10.1186/1472-6963-13-S2-S4

**Published:** 2013-05-31

**Authors:** Kenneth Sherr, Fatima Cuembelo, Cathy Michel, Sarah Gimbel, Mark Micek, Marina Kariaganis, Alusio Pio, João Luis Manuel, James Pfeiffer, Stephen Gloyd

**Affiliations:** 1 Department of Global Health, University of Washington, Seattle, USA; 2 Health Alliance International, Beira, Mozambique; 3 Community Health Department, School of Medicine, Eduardo Mondlane University, Maputo, Mozambique; 4 Sofala Provincial Health Directorate, Ministry of Health, Beira, Mozambique; 5 Beira Operations Research Center, Ministry of Health, Beira, Mozambique

## Abstract

**Background:**

Large increases in health sector investment and policies favoring upgrading and expanding the public sector health network have prioritized maternal and child health in Mozambique and, over the past decade, Mozambique has achieved substantial improvements in maternal and child health indicators. Over this same period, the government of Mozambique has continued to decentralize the management of public sector resources to the district level, including in the health sector, with the aim of bringing decision-making and resources closer to service beneficiaries. Weak district level management capacity has hindered the decentralization process, and building this capacity is an important link to ensure that resources translate to improved service delivery and further improvements in population health. A consortium of the Ministry of Health, Health Alliance International, Eduardo Mondlane University, and the University of Washington are implementing a health systems strengthening model in Sofala Province, central Mozambique.

**Description of implementation:**

The Mozambique Population Health Implementation and Training (PHIT) Partnership focuses on improving the quality of routine data and its use through appropriate tools to facilitate decision making by health system managers; strengthening management and planning capacity and funding district health plans; and building capacity for operations research to guide system-strengthening efforts. This seven-year effort covers all 13 districts and 146 health facilities in Sofala Province.

**Evaluation design:**

A quasi-experimental controlled time-series design will be used to assess the overall impact of the partnership strategy on under-5 mortality by examining changes in mortality pre- and post-implementation in Sofala Province compared with neighboring Manica Province. The evaluation will compare a broad range of input, process, output, and outcome variables to strengthen the plausibility that the partnership strategy led to health system improvements and subsequent population health impact.

**Discussion:**

The Mozambique PHIT Partnership expects to provide evidence on the effect of efforts to improve data quality coupled with the introduction of tools, training, and supervision to improve evidence-based decision making. This contribution to the knowledge base on what works to enhance health systems is highly replicable for rapid scale-up to other provinces in Mozambique, as well as other sub-Saharan African countries with limited resources and a commitment to comprehensive primary health care.

## Background

Sofala Province, located in central Mozambique, has a relatively high-population density and an estimated population of more than 1.8 million, 37% of which is urban [1]. Nearly 62% of the population is concentrated along the major shipping and transport route connecting the sea port of Beira to Zimbabwe. Of the 11 provinces in Mozambique, Sofala ranks among the poorest, though key health indicators are above the country average and have improved at a more accelerated pace than other provinces. Latest estimates show that the infant mortality rate is 81 per 1,000 live births, and the under-5 mortality rate is 134 per 1,000 live births, compared with 107 per 1,000 live births and 157 per 1,000 live births nationally [2]. Despite substantial reductions in mortality indices over the past decade, the four principal causes of under-5 mortality in Sofala remain diseases with available low-cost and effective prevention or treatment strategies, including malaria (32.9%), acute respiratory infections (10.9%), HIV (9.9%), and diarrheal diseases (8.9%) [3]. High levels of chronic (35.7%) and acute (7.4%) malnutrition result from persistent food insecurity [4]. Nationally, the maternal mortality ratio, which has seen a 50% reduction in the last decade, is still high at an estimated 490 per 100,000 live births [5].

Approximately 15.5% (over 185,000) adults aged 15-49 in Sofala Province are HIV-infected, with 60,000 estimated to be eligible for antiretroviral therapy (ART) [6]. Pediatric HIV infection is high, with an estimated 23,800 HIV-infected children in 2007 [7]. Tuberculosis (TB) incidence is estimated at more than 400 per 100,000 people, and HIV co-infection is common, with more than 60% of TB patients in Sofala also testing positive for HIV [8].

### Health systems coverage

Despite the high burden of disease, the use of formal health services provided through the National Health Service (NHS) remains high, particularly for basic preventive and curative maternal, newborn, and child health services.

Coverage of antenatal care services (at least one visit) is high in Sofala and has increased from 82% to 95% in the past decade. In the same time period, the institutional birth rate increased from 51% to 71%. Both measures are higher than the national average [4,9]. Of those receiving routine antenatal care services, more than 90% received syphilis testing and treatment, 46% received at least one dose of intermittent preventive treatment for malaria [10], and 74% received testing for HIV, reflecting variations in the delivery of effective interventions through the antenatal care platform [2]. Five hospitals in the province are equipped to perform cesarean births, and they perform these at a low rate of 2% of estimated births. Modern contraceptive use is low in Sofala among women of reproductive age (8%) and lower than the national average (12%).

Immunization coverage in Sofala Province is high, with 85% of children 12-23 months having received diphtheria-tetanus-pertussis (DTP3) vaccine, 87% measles vaccine, and 78% receiving all required vaccinations. An estimated 25% of children under 5 sleep under insecticide treated bednets [10]. Utilization of formal health services among symptomatic children under-5 is also high and above the national average, including among those with diarrheal symptoms (71.2% in Sofala compared with 58.5% nationally) and fever (75.4% compared to 58.8% nationally) [4]. Basic ambulatory care services are broadly available throughout the province and nearly 3 million people are reached annually through outpatient visits [11]. Integration of HIV care into outpatient services at smaller health centers has improved adult and pediatric access to HIV care, and in 2012 more than 27,300 people (nearly 50% of ART eligible patients) are on ART, including more than 2,800 children under 15 [12]. Case detection of new sputum smear positive pulmonary TB cases is estimated at 88.3%, and 83% of those initiating TB treatment are treated to completion [13]^.^

### The Mozambique National Health Service

Since its inception in 1975 following national independence, the Mozambique National Health Service (NHS) has rapidly expanded primary health care (PHC) services through a widespread network of health facilities. As a result of this expansion, the NHS is the major provider of formal health care services in Mozambique, providing 98% of outpatient services in Sofala Province.

The health network in Sofala Province includes 146 health facilities within its 13 districts, translating to a health facility to population ratio of 1/12,000 [14]. This network is organized into four basic levels of care, including 1) one quaternary-level hospital in Beira; 2) four secondary-level rural hospitals; 3) 114 urban and rural health centers; and 4) 27 health posts [11]. Over the past decade, the Mozambique Ministry of Health has prioritized expanding the overall number of facilities and enhancing facility capacity by transforming lower level health posts into health centers and increasing rural and district hospitals. Over the last decade, economic growth and increased development assistance has led to dramatic health sector spending increases, growing from less than USD$10 per capita in 2001 to approximately US$26 per capita in 2008, of which more than 70% is financed by external aid [15,16].

**Figure 1:**
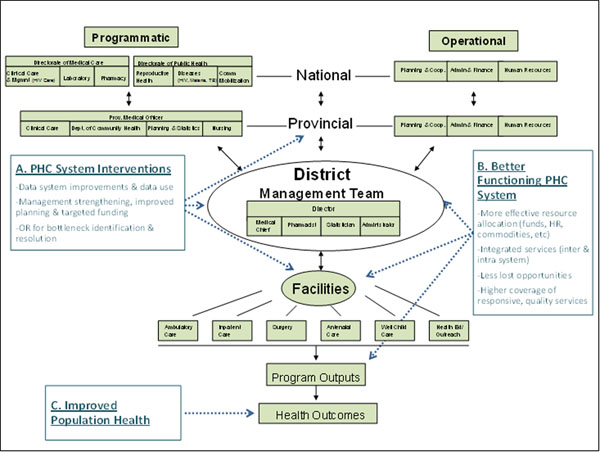
Simplified functional representations of PHC and support systems.

At the national level the Ministry of Health sets country health policy and manages both health programs and operational support services, including procurement and distribution of medicines and medical supplies to the provincial level (Figure 1). Each of Mozambique’s 11 provinces has its own health directorate that also performs operational and programmatic management functions and represents a key organizational unit through which primary health care services are managed, coordinated, and brought to scale. Each district health directorate has a management team comprised of a district director, chief medical officer, pharmacist, statistician and administrator, which is responsible for providing support for, and managing, health facilities that, in turn, provide primary health care services.

The Mozambique health system’s current decentralization process has moved important management and planning tasks from the provincial level to the district level. District management teams have become the vital link in the NHS to improve integrated care. Duties of these teams include planning, budgeting, human resources management, medical supply distribution, supervision, and data collection.

Despite Mozambique’s successes in improving the health care infrastructure and the high utilization of primary health care services, chronic resource shortages, vertical funding, and management challenges limit service coverage and quality. Mozambique ranks among the highest human resource-constrained countries in the world, with 2.4 doctors and 21 nurses/100,000 people; in Sofala, a population of 1.8 million, there are just 2,400 technical health workers and 2,400 support staff [17]. District health directorates also remain underfunded with limited technical, managerial, and workforce capacity to assume newly devolved responsibilities. District management is further hampered by a combination of weak data collection systems and limited capacity to analyze data for district level decision making and planning.

### District management and health systems strengthening

Government decentralization has become a cornerstone of public sector reform in Mozambique and in other low- and middle-income countries (LMICs), including in the health sector. The decentralization agenda has been promoted by multi and bilateral donors as a strategy to bring decision making closer to the people,[18-21] and has been sequentially implemented over the past decade [22,23]. District directorates are the health system unit that will determine the success of health sector decentralization. As described above, challenges remain in Mozambique to sufficiently support and build district management capacity for leadership, planning, resource allocation, and financial management. This district level capacity is essential for improving service delivery and quality at the facility level, and, ultimately, population health.

There is little evidence, however, on effective approaches to strengthening district-level management capacity. Fundamental to achieving this goal is the establishment of robust health information systems (HIS) and the capacity to use routine data for decision making. Approaches to improving HIS quality and consistency have been developed and are being implemented in LMICs, including in Mozambique [24-28]. Efforts have also been made to link HIS with improved service delivery, primarily focused on disease-specific services within limited geographic areas [29,30]. However, there is little evidence on successful efforts to improve data utilization among district managers and broadly across large health systems. This lack of acting on data is a critical gap in achieving better health outcomes. Research is needed on what works to increase the use of evidence for decision making and the rational use of scarce resources.

### Mozambique Population Health Implementation & Training (PHIT) Partnership

The PHIT Partnership aims to improve health system capacity and the functioning of the primary health care system – including more effective resource allocation, better integration of services, and high coverage of quality services – across the provincial, district, and facility levels in Sofala Province by focusing on strengthening data systems, management, and bottleneck resolution (Figure 1). Considering Sofala’s expansive primary health care network and high service utilization, it is envisioned that these improvements in health system performance will result in better health outcomes, including reduced child mortality.

District management teams are an essential link to achieving improvements in health systems performance and population health. With better training and new tools, managers can approach their operations as interconnected systems, focusing on facility-level and service-sector indicators, rather than isolated indicators of disease-based vertical programs. By considering the inputs and outputs of the entire health care system, this management approach facilitates the expansion of integrated PHC at the facility level and reduces service delivery gaps that occur if services are organized on disease-based classifications. Strengthening district level management capacity across Sofala’s 13 districts is a fundamental strategy to achieving the aim of the PHIT Partnership in Mozambique.

The partnership objectives are synergistic and include:

1. Improve the quality of routine data and develop appropriate tools to facilitate decision-making for provincial and district managers;

2. Strengthen integrated health systems management and planning in Sofala at district and provincial levels;

3. Build capacity and carry out innovative operations research (OR) to guide integration and system strengthening efforts.

## Description of implementation

A hallmark of the Mozambique PHIT Partnership is its integration into the Ministry of Health’s management structure at the provincial level, which is designed to facilitate the sustainability and scalability of its activities. Partnership technical personnel work within MOH offices to build the capacity of staff from operational and program sectors in conducting routine management activities rather than developing parallel systems. This integrated approach is designed to enable uptake of new ideas and methodologies from the PHIT Partnership into the health system, leading to rapid broad-scale and sustainable implementation of activities across Sofala Province.

Mozambique PHIT partners include the Sofala Provincial Health Directorate, Health Alliance International (HAI), the Ministry of Health’s Beira Operations Research Center (CIOB), Eduardo Mondlane School of Medicine (UEM), and the University of Washington (UW) departments of Global Health and Industrial & Systems Engineering. These institutions have more than 25 years of collaborative experience in central Mozambique. With technical support from HAI advisors, provincial health authorities are responsible for designing and implementing the PHIT model. The Beira Operations Research Center, an applied research arm of the Mozambique Ministry of Health’s National Health Institute, supports OR in the province and for the PHIT project; it was developed in 2007 with financial and technical support from Health Alliance International and UW. UEM faculty co-lead the project, train and mentor master’s-level students from Sofala Province, and provide technical support for associated applied research in Sofala. UW faculty support implementation research and lead evaluation efforts.

**Figure 2:**
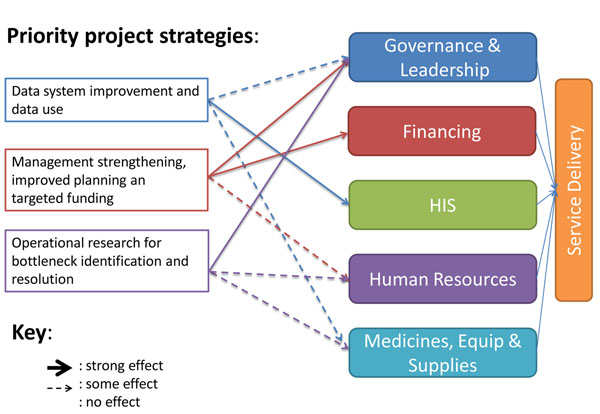
Priority strategies and expected effects on health system building blocks.

Given the integrated nature of the partnership approach, the project objectives are designed to lead to improvements across all six health system building blocks identified by WHO (Figure 2) [31]. However, partnership activities (described below by objective) are expected to lead to more pronounced improvements in the building blocks of governance and leadership, health information systems, and financing.

### Objective 1: Improve the quality of routine data and develop appropriate tools to facilitate decision making for provincial and district managers

The Mozambique PHIT Partnership endeavors to improve data quality through continual assessment of the availability, consistency, accuracy, and validity of data for key PHC system inputs from the HIS, and reinforcement of data quality feedback loops (Table 1). District and provincial HIS staff check data reports monthly for inconsistencies, and missing data and rapid feedback is given to correct data system problems. Using a data quality assurance assessment (DQA) model adapted from the Global Fund for use in Sofala Province, annual DQA assessments are carried out to assess HIS functioning at the provincial, district, and facility levels for key primary health care indicators building up from a sample of 27 health facilities from all districts in Sofala Province [24]. These 27 facilities were purposively chosen to include equal numbers of facilities from all 13 districts plus the quaternary-level referral hospital in the province, and to represent all facility levels in the Mozambique NHS. To translate these assessment activities into a strengthened HIS, partnership advisors work with health system counterparts to troubleshoot and resolve problems, primarily through feedback loops provided to facility and district managers during regular supervision visits as well as through facility level HIS report cards (See Additional File 1). Furthermore, the partnership supports updating HIS activities across multiple programs and sectors through training, supervision, and equipment purchase and maintenance (e.g., the expansion of the new Internet-linked, electronic pharmacy stock management system from the provincial to district pharmacy warehouses).

**Table 1:** Routine data system assessment approach

Type of evaluation	Method	Periodicity
Reliability / consistency	Evaluate stability (outliers, erratic data patterns) of facility-based reporting statistics over time	Monthly
	
	Check consistency of data from facility-based paper records with data in computerized HIS and data sent to provincial and national levels	Yearly (sample of 27 facilities, all districts)

Validity	Compare HIS data to community survey data (DHS, MICS)	Timed with community surveys

The PHIT Partnership has developed and disseminated simple and appropriate tools that summarize key indicators at facility, district, and provincial levels. These tools include a quarterly report card/data dashboard that provides longitudinal comparisons of key indicators across all facilities within a district, or across all districts within the province (See Additional File 2 for sample feedback tool). Additional job aids, such as supervision tools and checklists, have been developed and implemented through the partnership, which are routinely used by managers to improve the collection and use of health systems performance measures. Finally, a simple optimization model has been developed to simulate and improve human resource allocation. This Excel^®^-based tool is designed to identify inefficiencies and inequities in current personnel allocation and guide improved deployment processes at the district level.

### Objective 2: Strengthen integrated health systems management and planning in Sofala at district and provincial levels

Partnership activities are designed to promote data-driven decision making by building capacity in management and leadership through a mix of in-service trainings and post-training coaching. In-service training courses for health managers are based on a MOH training curriculum on using data for decision making, including program monitoring as a means of identifying priority problems, linking service utilization patterns with resource planning, and evaluating small-scale service delivery [32]. District and facility health managers receive ongoing post-training coaching via quarterly supportive supervision visits from provincial and district health systems managers, and from ongoing mentorship from partnership teams embedded in the provincial health department. Supporting the quality and frequency of province-to-district and district-to-facility supervision visits is essential for ensuring systems accountability and overcoming many of the management-related health system deficiencies [33].

Routine district-level meetings involving health facility directors, district and provincial managers, and partnership staff are held to jointly review program activities, identify performance gaps, plan solutions, and follow-up on previously identified problems and implementation of proposed solutions. Partnership resources are also allocated to support the development and implementation of annual costed health plans for districts and hospitals with independent budget authority. Funding for district plan implementation is provided in accordance with routine MOH financial management practices; funding is based on population size, health service capacity, service utilization, and priorities developed by district management teams using partnership-designed decision-making tools.

### Objective 3: Build capacity and carry out innovative OR to guide integration and system- strengthening efforts

Sources of service delivery bottlenecks and potential solutions are often not fully understood or agreed upon by district and provincial managers. The partnership aims to build capacity to undertake simple, applied systems analysis and other OR at the district and facility levels to guide service integration efforts; increase identification of modifiable service-delivery barriers; and evaluate the effectiveness of potential innovations before scaling up throughout the province.

The partnership provides technical and financial support for the Ministry of Health’s Sofala-based Beira Operations Research Center, master’s level public health training at the Eduardo Mondlane University, and applied thesis work for public health trainees. Targeted quantitative and qualitative OR projects are designed and implemented with participation of health system managers at the district and provincial levels to uncover reasons for systems bottlenecks, and to develop appropriate solutions that are likely to have a large impact on health system functioning. Examples of two ongoing OR projects include evaluating the impact of introducing shifts for outpatient care to extend working hours at a large urban clinic on health service utilization and the application of process mapping and subsequent iterative quality improvement efforts to reduce loss to follow-up among HIV-infected children.

### Geographic scope of Mozambique PHIT Partnership

**Figure 3:**
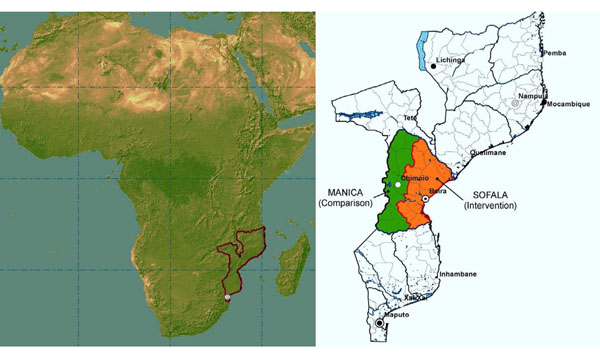
Map of intervention and comparison provinces.

The partnership implementation area covers all of Sofala Province, including the provincial health directorate, 13 districts, and 146 health facilities (Figure 3). Sofala was chosen as the focal region based on the extensive collaborative history of the lead partners in the province. The PHIT design is focused on an entire province because it is an important administrative unit in the Mozambique health system and provincial capacity must be considered in any approach that aims to sustainably improve district (and, subsequently, health facility) capacity. Furthermore, all districts in the province were selected for the Mozambique PHIT Partnership to provide a comprehensive understanding of the partnership effect on health management capacity across heterogeneous districts. Such an understanding is important to determining which aspects of the partnership design are most suited for national scale-up and which may need modification to fit local contexts.

The comparison area selected for the impact evaluation is neighboring Manica province. Manica was chosen because it is the most similar province in Mozambique to Sofala in terms of population size (1.7 vs*.* 1.8 million), number of districts (10 vs. 13), number of health facilities (84 vs. 146), baseline health measures, and culture.

### Mid-stream adaptations

Because of shifts in national programs, available funding, and iterative learning, there have been a number of notable changes to the PHIT intervention design over time. National changes such as new registries and data reporting systems for maternal and reproductive health services and HIV care and treatment have created new training needs that the PHIT Partnership has accommodated.

Due to the finalization of a national training model for improving data quality and linking data with decision making (including activity planning and budgeting), this module has become a core component of the partnership intervention. Though this training module is being introduced nationwide, PHIT project resources has accelerated its introduction in Sofala Province to reach district-level managers. Iterative appraisals of data collection procedures and dissemination tools (such as the routine performance dashboards and supervision guides) have led to their continued modification over the course of the study period. In addition, due to changes in complementary funding and to better align the technical assistance approach, the project technical assistance model was adapted to focus on one team of advisors based at the provincial level, rather than five teams based sub-provincially with responsibility for supporting multiple districts.

### Timetable of activities

**Figure 4:**
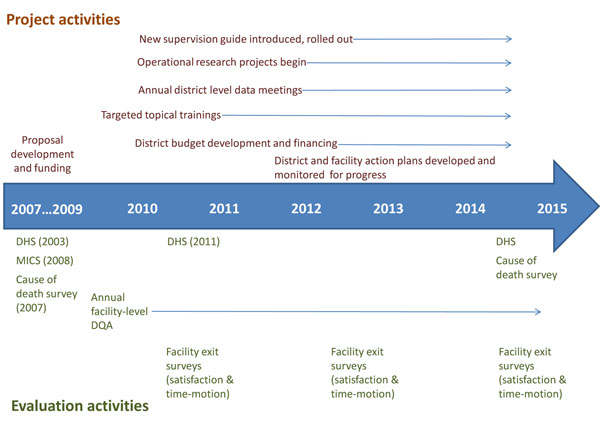
Timetable for partnersip implementation and evaluation.

Implementation of the Mozambique PHIT strategy covers a seven-year period beginning July 01, 2009 (Figure 4). Implementation of the package of PHIT interventions was initiated after a short design period in 2009, and will wind down in year six of the partnership. Monitoring and evaluation activities span the entire seven year implementation period.

## Evaluation design

### Study design

A quasi-experimental controlled time-series design will be used to assess the overall impact of the partnership strategy on under-5 mortality by examining changes in mortality pre- and post-implementation of the PHIT strategy in Sofala (intervention province) compared with Manica (comparison province), using a difference-in-differences analysis approach taking into account potential confounders using regression techniques. Analyses will be conducting using Stata 11 (College Station, TX).

The impact evaluation will also explore the plausibility that our priority strategies were associated with positive changes in intermediate outcomes. Trends will be analyzed for three types of indicators over the PHIT implementation period: 1) health system responsiveness indicators, including patient satisfaction, wait times, consult times, tracer medicine, commodity availability, and data system quality measured via health facility surveys and observations; 2) health system coverage indicators, including contraceptive prevalence rate, antenatal care, intermittent preventive treatment of malaria during pregnancy, skilled birth attendant, DTP3, indicators on the correct management of childhood illnesses, vitamin A supplementation, and children under 5 sleeping under an insecticide treated bednet measured via national demographic and health surveys (DHS) and multi-indicator cluster surveys (MICS); and 3) health status indicators, including child mortality, child nutrition (stunting and wasting), and total fertility rate, also measured via DHS/MICS surveys. A full list of core indicators is available in the evaluation framework paper in the series [34].

Program documentation data will be summarized at the district level and over time to provide a clear indication of the intensity and quality of implementation. Data on intermediate outcomes (quality, utilization, coverage) will be summarized, and levels and changes over time will be compared for the intervention and comparison provinces. Some health system readiness indicators (tracer medicine and commodity availability, as well as data system quality) will be available only in the intervention areas, but examining levels and trends will assist us in determining the intensity and quality with which partnership interventions were implemented. Results of verbal autopsies will be used to assess whether changes in the cause of child death structure are consistent with the patterns of coverage for interventions targeting specific diseases. We will summarize all data using the Partnership Impact Model, and, where possible, use multivariate analysis. Preliminary results of the plausibility analysis will be reviewed with Ministry of Health and other stakeholders for help in the interpretation of the results.

### Sample size/power calculation

Because the DHS/MICS sample sizes are defined by the Mozambique Ministry of Health and survey partners, we are able to detect a determined level of difference between intervention and comparison provinces assuming 80% power and α = 0.05. Assuming that the sample sizes for the 2016 DHS will be comparable to the 2008 MICS, the provided sample size will be adequate to detect a change of <10% in core coverage and impact indicators pre- vs. post-intervention in Sofala Province (detectable alternative values were estimated using PS Power and Sample Size Calculator, version 3.0, January 2009, Dupont and Plummer) [34].

The sample size for the health facility exit surveys was determined based on a clustered analysis using each health facility consult-service pair as a separate cluster (13 clusters per province) and conservatively based on a post-only design between intervention and comparison facilities, assuming that addition of pre/post measurements will increase our power for the final analysis. Analyses were powered to detect the difference between an 83% patient satisfaction rate (rating 4 or 5 on a 5-point Likert scale) in comparison clusters and a 90% satisfaction rate in intervention clusters. To achieve 80% power with an α = 0.05 and a coefficient of variation between clusters (k) of 0.05, 62 patients are required per cluster. Anticipating non-response and missing values, a sample size of 975 patients per province per time point (75 patients per cluster) will be obtained.

### Data sources

Data on health care coverage and health status rely on DHS and MICS questionnaires, which are collected independently of the partnership. Indicators from these surveys will be at the provincial level, and include health status and coverage measures.

Patient satisfaction data are collected using a questionnaire developed for the partnership project based on a satisfaction questionnaire developed by the Mozambique Ministry of Health in 2009. Data on wait and consult times are collected via direct observation by study teams recording the times of patient events (arrival, entering consult room, exiting consult room) of consecutive patients over a four-day period of time at each of the 12 study facilities (six per province). Data on tracer medicines and commodities, as well as data quality, are generated from facility and district level surveys and direct observation from a sample of 27 facilities in the intervention province.

### Analysis approach

The difference-in-differences analysis relies on the comparison of measurements between the pre- vs. post-intervention periods in intervention and comparison provinces. The final analysis will include a regression model with dependent variables being the main outcome indicators in separate models, independent variables including pre/post-intervention, intervention/comparison province, year of measurement (for analyses with repeated measures), and the interaction between pre/post and intervention/comparison to determine whether there was a significant change related to the intervention compared to the pre-intervention phase and the control province. Individuals will be the unit of the analyses, and for patient satisfaction and time-motion indicators, we will additionally perform an analysis clustered by the primary sampling unit (health facility clinical service cluster).

### Economic assessment

Assessment of costs, including total spending and re-allocation of resources within health programs related to the activities of this partnership, are tracked as part of the economic assessment. These costs include an accounting of 1) annual government funding by subsystem (i.e., drug, human resources, lab, pharmacy), including both governmental resources and external aid channeled through governmental budgets; 2) annual external donor funding in Sofala, including partnership resources and other donor and NGO funding; and 3) annual out-of-pocket expenditures obtained from public sector revenue reports and community surveys. Indirect linkages will be drawn between total spending, spending in health system subcomponent areas, and changes in process measures, program outputs, and program outcomes.

### Preliminary results

Experience to date designing and implementing the evaluation approach has highlighted a number of challenges and opportunities. The impact evaluation relies largely on data from community surveys financed and implemented for use outside the partnership. Because these surveys are powered to the partnership’s geographic unit of focus – the provincial level – they are an effective, cost-saving evaluation approach that reserves partnership resources (financial, human, and logistical) for implementation and complimentary applied research efforts. Furthermore, using standardized community surveys at the country level reinforces their utility for country-owned evaluation. However, the use of these community surveys results in a number of evaluation constraints. For example, there is little flexibility in adapting evaluation measures, defining the survey implementation timeframe, or increasing sample size to meet PHIT-specific evaluation needs given that the baseline household survey was carried out prior to initiation of Mozambique PHIT activities. In some cases, the sample size may be insufficient to detect changes in less frequent outcome measures important for assessing the effect of the partnership activities on strengthening the health system and improving health. Another notable challenge is the pre-existing temporal trends in coverage and impact measures in both the intervention and comparison provinces, and the potential for contamination associated with the passive adoption of successful elements of the intervention in the comparison province, which may complicate attempts to demonstrate improvements attributable to the PHIT Partnership.

Despite these challenges, data collection plans are on track, creating opportunities for capacity building among partner institutions in Mozambique on conducting and analyzing rigorous and complex impact evaluations.

## Discussion

A number of lessons have been learned about building health system capacity from the early years of implementing PHIT Partnership activities in Mozambique. (See Table 2 for a description of the key success, challenges, and implementation adaptations that the investigators have observed thus far.) The first is that decentralization is not a linear or sequential process, and often it is not clear which activities are within the scope of control of district, provincial, or national managers. The lack of a defined and sequential blueprint for decentralization presents challenges for defining how and where to intervene to accelerate and strengthen this process. A response to the often lack of clarity, and an important element in supporting complex health systems, is to treat the health system in its entirety rather than focusing on the district or provincial levels alone. By building communication and support channels across all levels of the health system, essential buy-in from leaders at the provincial or district level can be more readily obtained and serve as a catalyst to rollout intervention efforts broadly across districts and facilities in the health system.

**Table 2:** Mozambique PHIT Partnership: Successes, Challenges, Adaptations

Successes
**Collaboration and ownership by provincial and district Ministry of Health authorities** Embedded technical and financial support has built ownership of the PHIT activities by health system leadership, which has led to refinements in PHIT tools to be contextually appropriate in design, accelerate the pace of implementation in the province, and provides an avenue for further scale-up. **Scale of implementation** Working across an entire province has directed support to resolve upstream and downstream health system bottlenecks, and reach a large scale of beneficiaries. **Development of sustainable applied research capacity** Project support for the MOH Beira Operations Research Center to meet implementation research and evaluation objectives has built sustainable capacity and led to new funding opportunities.

**Challenges**

**Management and Leadership training** Training curricula focused on capacity building for both management and leadership that are readily available, contextually appropriate and feasible to implement with limited time and resources are in short supply. **Complementary funding** Unpredictable changes in complementary funding have resulted in adaptations to the design and size of PHIT technical support personnel. **Slow, uneven decentralization process** The pace and process of public sector decentralization is unpredictable, challenging project assumptions on how to most define the role of district managers and strengthen their management capacity. **Turnover of key managers** Frequent turnover of district and provincial managers requires ongoing stakeholder engagement and flexibility in programming.

**Adaptations**

**Focus on practical management skills building approach** After initial trainings for district managers, management capacity support has focused on ongoing mentoring to put into action data driven decision making and system improvement capabilities. **Adaptations to the design and size of PHIT technical support personnel** The reconfigured design supports district capacity through teams based the provincial health directorate, rather than five sub-provincial teams that directly supported district managers across the 13 districts.

A second notable challenge has been the lack of resources in the NHS. One striking resource shortage has been in the area of workforce, and, in particular, district management teams. Because of the large deficit of human resources in the NHS, there are simply insufficient numbers of personnel to assign them to only taking on management roles. Rather, personnel are forced to assume multiple positions spanning both district and facility levels and involving both managerial and clinical responsibilities. District directors, chief medical officers, and statisticians, in particular, are often required to combine their positions with other tasks. Our experience is that personnel shortages and heavy workloads are a more important constraint than frequent turnover. Resource shortages experienced at health facilities throughout the province —including financial resources, medicines, and medical supplies — represent a continued and, at times, overwhelming challenge to the provision of quality health services.

The PHIT Partnership has also encountered challenges in building leadership capacity at scale, which requires an intensive process using skilled mentors to train individual health system managers. Using a practical, skills-based training approach in management has been a more feasible approach to reach across all 13 district management teams.

One unique element of the Mozambique PHIT Partnership has been its integration into the NHS management system. This approach facilitates the rapid scale-up of new ideas or improvements that are highly replicable and sustainable. However, flexibility has been required to allow sufficient time and opportunity for adaptation of the implementation approach and simplification of tools to meet the capacity and needs of the health system.

Despite the implementation challenges, the Mozambique PHIT Partnership expects to be able to demonstrate whether its approach to health systems strengthening leads to improvements in system processes and outputs, which in turn result in increases in coverage levels and a positive impact on population health. By providing evidence on the effect of efforts to improve data quality coupled with the introduction of tools, training, and supervision to improve evidence-based decision making, we expect to contribute to the knowledge base on what works to enhance health systems in an African context and potentially in other LMICs. Furthermore, we expect to contribute to the knowledge base regarding how to design and conduct impact evaluations for large-scale, complex interventions in real-world implementation settings.

## List of abbreviations used

ART: Antiretroviral therapy; CIOB: Beira Operatins Research Center; DHS: Demographic and health survey; DQA: Data quality assurance; DTP: Diphtheria, tetanus, and pertussis vaccine; HAI: Health Alliance International; HIS: Health information system; HIV: Human immunodeficiency virus; LMIC: Lower- and middle-income countries; MICS: Multi-indicator cluster survey; NGO: Non-governmental organization; NHS: National Health Service; OR: Operations research; PHC: Primary health care; PHIT: Population Health Implementation and Training; TB: Tuberculosis; UEM: Eduardo Mondlane University (Portuguese); UW: University of Washington; WHO: World Health Organization.

## Competing interests

The authors declare that they have no competing interests.

## Supplementary Material

Additional file 1 Click here for file

Additional file 2 Click here for file

## Declarations

This article has been published as part of *BMC Health Services Research* Volume 13 Supplement 2, 2013: Improving primary health care to achieve population impact: the African Health Initiative. The full contents of the supplement are available online at http://www.biomedcentral.com/bmchealthservres/supplements/13/S2. Publication of this supplement was supported by the African Health Initiative of the Doris Duke Charitable Foundation.
