# Supplementary material for: Strengthening integrated primary health care in Sofala, Mozambique
Source: BMC Health Serv Res. 2013 May 31;13(Suppl 2):S4. doi: 10.1186/1472-6963-13-S2-S4 (PMC3668215; doi:10.1186/1472-6963-13-S2-S4)
Supplement: Additional file 1 [file 1472-6963-13-S2-S4-S1.pdf]

DIRECÇÃO PROVINCIAL DE SAÚDE DE SOFALA

COMO ESTÃO OS DADOS NA SUA UNIDADE SANITÁRIA?

CIOB
CENTRO DE INVESTIGAÇÕES OPERACIONAIS DA BEIRA

O estudo "**Avaliação de quatro indicadores de rotina dos cuidados de saúde na província de Sofala**" tem como objectivo avaliar os dados de rotina de 2010 de quatro indicadores do Sistema de Informação da Saúde. Cada ano para os próximos quatro anos, uma equipe irá as mesmas U.S. para recolher os dados.

Recolheu-se dados de quatro indicadores dos cuidados primários de saúde do ano 2010:

1. Nº de partos institucionais
2. Nº de primeira consulta pré-natal
3. Nº de consultas externas
4. Nº de 3ª dose de DPT-HepB-Hib

Depois, comparou-se a concordância dos dados e fez-se um ordenamento das U.S. segundo a concordância de dados. As U.S. estão ordenadas numericamente de primeira a última posição.

2010		
**Distrito**	**Unidade Sanitária**	
Nhamatanda	CS Tica	1
Machanga	CS Inharingue	2
Muanza	CS Galinha	2
Beira	CS Chingussura	3
Caia	CS Murema	4
Marromeu	CS Chueza	5
Beira	CS Ponta Gêa	6
Chemba	PS Catulene	6
Chibabava	HR Muxunguê	7
Chibabava	CS Mangunde	7
Gorongosa	CS Mucodza	7
Búzi	CS Bândua	8
Chemba	CS Chemba	9
Cheringoma	CS Mazamba	10
Dondo	CS Savane	11
Nhamantanda	HR Nhamatanda	12
Cheringoma	CS Inhaminga	13
Maringuê	CS Canxixe	14
Búzi	HR Búzi	15
Machanga	CS Machanga	16
Marromeu	HR Marromeu	17
Dondo	CS Dondo	18
Gorongosa	CS Gorongosa	19
Caia	CS Caia	20
Maringuê	CS Maringuê	20
Muanza	CS Muanza	21

QUEM TEM OS MELHORES DADOS DE 2010?

CS DE TICA!!

QUAIS U.S. ESTÃO DE PARABÊNS EM 2010?

CS INHARINGUE
CS GALINHA
CS CHINGUSSURA
CS MUREMA

QUAIS U.S. PRECISAM TRABALHAR MUITO NOS SEUS DADOS?

CS DONDO
CS GORONGOSA
CS CAIA
CS MARINGUÊ
CS MUANZA

COMO PODEM MELHORAR OS DADOS?

- Melhorar o preenchimento do livro de registo de DPT-HepB-Hib
- Arquivar os livros de registo de forma segura e apropriada na U.S. num período de 5 anos
- As U.S. tem que ficar com cópias dos resumos mensais na US, especialmente os C.S. da sede e H.R.
- O NEP Distrital tem que sempre ficar com uma cópia dos dados de Módulo Básico num disco

AVALIAÇÃO DE QUATRO INDICADORES DE ROTINA DE CUIDADOS DE SAÚDE NA PROVÍNCIA DE SOFALA (2009-2010)

Roxanne Hoek, Titos Quembo, Pires Afonso, Victoria Porthé, Catherine Michel, Julie Cliff, Fátima Cuembelo, Sarah Gimbel, Kenneth Sherr, Aluísio Gonzaga Pio, Francisco Macuácua, João Luís Manuel, Marina Karigianis

CIOB
CENTRO DE INVESTIGAÇÕES OPERACIONAIS DA BEIRA

Introdução: Este estudo faz parte de um projecto composto por uma parceria de sete anos (2009-2016) entre a Fundação de Caridade Doris Duke (DDCF), Ministério da Saúde (MISAU), Universidade Eduardo Mondlane (UEM), Universidade de Washington (UW) e Health Alliance International (HAI). A parceria tem como objectivo fortalecer os cuidados primários de saúde na província de Sofala. **O objectivo geral** do estudo é: Avaliar os dados de rotina de 2009 e 2010 de quatro indicadores do Sistema de Informação da Saúde.

Material e Métodos: Estudo retrospectivo transversal. Recolheu-se dados de quatro indicadores dos cuidados primários de saúde dos anos 2009 e 2010:

1. Nº de partos institucionais
2. Nº de primeira consulta pré-natal
3. Nº de consultas externas
4. Nº de 3ª dose de DPT-HepB-Hib

Os dados foram recolhidos em três níveis do serviço nacional de saúde: Unidade Sanitária (US), Serviço Distrital de Saúde, Mulher e Acção Social (SDSMAS) e a Direcção Provincial de Saúde de Sofala (DPS). Foram seleccionadas 27 US, duas US por cada distrito da província e Hospital Central da Beira. Calculou-se a percentagem de disponibilidade de dados mensais, que significa instrumentos encontrados durante a recolha de dados. Através dum padrão de comparação, fez-se a análise de concordância dos dados. Dividiu-se os meses com dados com margem de erro inferior a 10% pelos meses disponíveis. O resultado representa a percentagem de dados de alta concordância.

Percentagem Global de Disponibilidade e Concordância Alta, 2009-2010

Indicadores	2009	2010	Instrumentos	2009	2010
**Partos Institucionais**			**Livro de Registo**		
Disponibilidade	88,0%	89,9%	Disponibilidade	84,5%	94,3%
Alta Concordância	87,2%	93,0%			
			**Resumo Mensal da US**		
			Disponibilidade	78,6%	73,4%
**Consultas Externas**			Alta Concordância *(com livro de registo)*	49,6%	53,1%
Disponibilidade	76,2%	89,0%			
Alta Concordância	61,4%	72,9%	**Resumo Distrital em Papel**		
			Disponibilidade	64,1%	91,7%
			Alta Concordância *(com resumo mensal da US)*	88,3%	93,1%
**1ª Consulta Pré-natal**					
Disponibilidade	83,1%	85,8%	**Resumo Distrital Electrónico**		
Alta Concordância	87,8%	91,5%	Disponibilidade	97,7%	91,4%
			Alta Concordância *(com resumo mensal da US)*	87,0%	91,1%
**3ª Dose DPT-HepB-Hib**			**Resumo Provincial Electrónico**		
Disponibilidade	89,5%	89,6%	Disponibilidade	99,4%	98,9%
Alta Concordância	71,5%	71,6%	Alta Concordância *(com resumo mensal da US)*	89,0%	92,3%
**Total**					
**Disponibilidade**				**84,3%**	**88,5%**
**Alta Concordância**				**78,4%**	**82,1%**

Conclusão: De forma agregada, houve aumento ligeiro da disponibilidade e concordância dos dados em 2010. Especialmente preocupante é a baixa qualidade de dados ao nível da US, uma vez que é a base (fonte primária) onde se criam os dados. Em particular, precisa-se enfocar nos dados de DPT-HepB-Hib e consultas externas na US.

Recomendações:

US: Melhorar o preenchimento do livro de registo de DPT-HepB-Hib; Arquivar os livros de registo de forma segura e apropriada na US num período de 5 anos; Todas as US tem que ficar com cópias dos resumos mensais na US, especialmente os CS da sede e HR

SDSMAS/DPS: O NEP Distrital tem que sempre ficar com uma cópia dos dados de Módulo Básico num disco; DPS e os SDSMAS deveriam dirigir as suas intervenções dando prioridade as US, especialmente aos HR e CS Tipo I; DPS deveria melhorar a provisão e gestão dos instrumentos utilizados pelo SIS para evitar as ropturas de stock

Encontre a sua Unidade Sanitária na tabela para ver a progressão de 2009 para 2010!

A tabela mostra o ordenamento das US segundo a disponibilidade e concordância de dados. A mesma está dividida em dois indicadores.
Para a disponibilidade, as US estão organizadas de maior a menor percentagem de disponibilidade. Para a concordância, as US estão ordenadas numericamente de primeira a última posição.

Partos Institucionais							
% de Disponibilidade				Concordância			
2009		2010		2009		2010	
CS Caia	100%	CS Chingussura	100%	1	CS Mazamba	1	HR Nhamatanda
PS Catulene	100%	CS Bândua	100%	2	HR Muxunguê	1	CS Tica
CS Mazamba	100%	CS Chemba	100%	2	CS Canxixe	2	CS Bândua
HR Muxunguê	100%	PS Catulene	100%	3	PS Catulene	2	CS Mazamba
CS Mangunde	100%	CS Mazamba	100%	3	HR Marromeu	2	HR Muxunguê
CS Canxixe	100%	HR Muxunguê	100%	4	HR Búzi	2	CS Inharingue
HR Búzi	98.3%	CS Inharingue	100%	4	CS Maringuê	3	CS Mangunde
CS Bândua	98.3%	CS Ponta Gêa	98.3%	4	CS Chueza	4	CS Ponta Gêa
CS Mucodza	98.3%	CS Mangunde	98.3%	5	CS Bândua	4	CS Chemba
CS Maringuê	98.3%	CS Savane	98.3%	6	CS Chemba	4	CS Mucodza
HR Marromeu	98.3%	HR Nhamatanda	98.3%	6	CS Inhaminga	5	CS Chingussura
CS Galinha	98.3%	CS Tica	98.3%	7	CS Mangunde	5	CS Murema
CS Inhaminga	96.7%	CS Murema	96.7%	7	CS Mucodza	5	CS Galinha
CS Chueza	96.7%	CS Chueza	96.7%	7	CS Galinha	6	PS Catulene
CS Murema	95.0%	CS Galinha	96.7%	8	CS Inharingue	7	CS Savane
CS Chemba	91.7%	CS Mucodza	93.3%	9	CS Tica	7	CS Chueza
CS Muanza	90.0%	CS Inhaminga	80.0%	10	CS Murema	8	CS Canxixe
CS Inharingue	85.0%	CS Dondo	80.0%	10	HR Nhamatanda	9	HC Beira
CS Savane	80.0%	CS Canxixe	80.0%	11	CS Ponta Gêa	10	CS Maringuê
CS Gorongosa	80.0%	HC Beira	78.3%	12	CS Savane	11	HR Búzi
CS Tica	80.0%	CS Caia	78.3%	13	CS Chingussura	12	CS Gorongosa
HR Nhamatanda	75.0%	CS Machanga	78.3%	14	HC Beira	13	CS Caia
CS Ponta Gêa	68.3%	CS Maringuê	78.3%	15	CS Muanza	13	CS Inhaminga
CS Chingussura	63.3%	HR Marromeu	78.3%	16	CS Dondo	13	CS Dondo
CS Dondo	63.3%	CS Gorongosa	75.0%	17	CS Caia	13	CS Machanga
HC Beira	60.0%	CS Muanza	75.0%	18	CS Gorongosa	13	HR Marromeu
CS Machanga	60.0%	HR Búzi	71.7%	18	CS Machanga	13	CS Muanza

Consultas Externas							
% de Disponibilidade				Concordância			
2009		2010		2009		2010	
CS Chemba	98.3%	CS Inhaminga	100%	1	CS Inharingue	1	CS Inharingue
CS Bândua	90.0%	HR Muxunguê	100%	2	CS Mazamba	2	CS Galinha
HR Búzi	86.7%	CS Mangunde	100%	2	CS Savane	3	CS Murema
CS Dondo	86.7%	CS Muanza	100%	2	CS Galinha	3	CS Mazamba
HR Muxunguê	83.3%	CS Ponta Gêa	98.3%	3	CS Mangunde	3	CS Savane
CS Mangunde	83.3%	CS Chingussura	98.3%	4	CS Mucodza	3	CS Tica
HC Beira	80.0%	PS Catulene	98.3%	5	CS Chemba	4	CS Chueza
CS Mazamba	80.0%	CS Inharingue	98.3%	6	CS Tica	5	PS Catulene
CS Inhaminga	80.0%	CS Galinha	98.3%	7	CS Bândua	6	CS Inhaminga
CS Inharingue	80.0%	CS Tica	98.3%	7	PS Catulene	7	HR Muxunguê
CS Maringuê	80.0%	CS Mazamba	96.7%	7	HR Muxunguê	8	CS Mangunde
CS Galinha	80.0%	CS Chueza	96.7%	7	CS Machanga	9	CS Chingussura
CS Savane	78.3%	HR Nhamatanda	96.7%	8	CS Muanza	9	CS Mucodza
CS Machanga	78.3%	CS Savane	95.0%	9	CS Chueza	9	CS Muanza
CS Muanza	78.3%	CS Murema	91.7%	10	CS Dondo	10	CS Canxixe
HR Nhamatanda	78.3%	HR Búzi	90.0%	11	CS Chingussura	11	CS Bândua
CS Chingussura	76.7%	CS Mucodza	90.0%	11	HR Búzi	12	HR Nhamatanda
PS Catulene	76.7%	CS Bândua	80.0%	12	HR Nhamatanda	13	HC Beira
CS Mucodza	76.7%	CS Chemba	80.0%	13	CS Gorongosa	14	CS Ponta Gêa
CS Chueza	75.0%	CS Machanga	80.0%	14	CS Ponta Gêa	15	HR Búzi
CS Gorongosa	73.3%	HR Marromeu	80.0%	14	HC Beira	15	CS Caia
CS Ponta Gêa	71.7%	CS Dondo	78.3%	14	CS Murema	15	CS Chemba
CS Tica	66.7%	CS Canxixe	78.3%	14	CS Caia	15	CS Dondo
CS Murema	61.7%	CS Caia	76.7%	14	CS Inhaminga	15	CS Gorongosa
HR Marromeu	60.0%	HC Beira	71.7%	14	CS Maringuê	15	CS Machanga
CS Caia	48.3%	CS Gorongosa	70.0%	14	CS Canxixe	15	CS Maringuê
CS Canxixe	48.3%	CS Maringuê	60.0%	14	HR Marromeu	15	HR Marromeu

Encontre a sua Unidade Sanitária na tabela para ver a progressão de 2009 para 2010!

A tabela mostra o ordenamento das US segundo a disponibilidade e concordância de dados. A mesma está dividida em dois indicadores.
Para a disponibilidade, as US estão organizadas de maior a menor percentagem de disponibilidade. Para a concordância, as US estão ordenadas numericamente de primeira a última posição.

1as CPN								DPT3-HepB-Hib							
% de Disponibilidade				Concordância				% de Disponibilidade				Concordância			
2009		2010		2009		2010		2009		2010		2009		2010	
CS Mazamba	100%	CS Chemba	100%	1	CS Mazamba	1	CS Galinha	HR Búzi	100%	CS Ponta Gêa	100%	1	HR Marromeu	1	CS Chemba
CS Chemba	98.6%	CS Inharingue	98.6%	2	HR Muxunguê	2	CS Chueza	CS Chueza	100%	CS Murema	100%	1	HR Nhamatanda	2	CS Ponta Gêa
CS Canxixe	98.6%	CS Chueza	98.6%	3	CS Galinha	2	CS Tica	CS Bândua	98.6%	CS Chemba	100%	2	CS Tica	2	CS Chingussura
CS Galinha	98.6%	CS Galinha	98.6%	4	CS Murema	3	HR Muxunguê	CS Mazamba	98.6%	CS Inhaminga	100%	3	CS Caia	2	CS Inhaminga
HR Muxunguê	97.2%	CS Bândua	97.2%	4	HR Marromeu	4	CS Ponta Gêa	HR Marromeu	98.6%	CS Dondo	100%	3	CS Mazamba	3	CS Machanga
CS Mangunde	97.2%	PS Catulene	97.2%	5	CS Bândua	4	CS Murema	HR Nhamatanda	98.6%	CS Machanga	98.6%	4	CS Chueza	4	CS Mucodza
CS Mucodza	95.8%	HR Muxunguê	95.8%	5	CS Canxixe	5	CS Chingussura	CS Tica	98.6%	HR Muxunguê	97.2%	4	CS Muanza	4	HR Marromeu
CS Murema	94.4%	CS Mangunde	95.8%	6	CS Chemba	5	CS Bândua	CS Inharingue	95.8%	CS Mucodza	95.8%	5	HR Búzi	5	CS Dondo
CS Muanza	94.4%	CS Mucodza	95.8%	6	CS Mangunde	5	PS Catulene	CS Mucodza	94.4%	CS Inharingue	95.8%	6	CS Bândua	6	HR Búzi
HR Búzi	93.1%	CS Tica	95.8%	7	CS Mucodza	6	CS Mangunde	CS Maringuê	94.4%	CS Chingussura	94.4%	6	CS Mucodza	6	HR Nhamatanda
CS Inhaminga	90.3%	CS Murema	94.4%	8	CS Caia	6	CS Inharingue	CS Muanza	94.4%	CS Galinha	94.4%	6	CS Maringuê	7	CS Caia
CS Caia	88.9%	CS Mazamba	91.7%	8	CS Maringuê	7	CS Chemba	HR Muxunguê	93.1%	HR Búzi	90.3%	7	CS Inharingue	8	CS Bândua
CS Savane	88.9%	HR Búzi	90.3%	9	CS Savane	8	CS Mucodza	CS Caia	91.7%	PS Catulene	90.3%	8	CS Chemba	8	PS Catulene
CS Bândua	84.7%	CS Gorongosa	87.5%	9	CS Dondo	9	CS Canxixe	CS Savane	91.7%	CS Chueza	90.3%	8	CS Mangunde	8	CS Mangunde
HR Marromeu	80.6%	CS Ponta Gêa	81.9%	10	CS Inhaminga	10	CS Savane	CS Galinha	91.7%	CS Mazamba	88.9%	8	CS Dondo	9	CS Inharingue
CS Chueza	79.2%	CS Caia	80.6%	11	CS Tica	11	CS Gorongosa	CS Machanga	88.9%	CS Tica	88.9%	9	CS Ponta Gêa	9	CS Tica
CS Gorongosa	77.8%	CS Inhaminga	80.6%	12	HR Búzi	12	HR Búzi	CS Murema	87.5%	HR Nhamatanda	87.5%	10	CS Galinha	10	CS Murema
CS Dondo	75.0%	CS Canxixe	80.6%	13	CS Ponta Gêa	12	CS Mazamba	CS Dondo	87.5%	CS Bândua	83.3%	11	CS Chingussura	10	CS Chueza
CS Inharingue	73.6%	CS Muanza	80.6%	13	CS Inharingue	13	CS Maringuê	CS Inhaminga	84.7%	CS Mangunde	83.3%	11	HR Muxunguê	10	CS Galinha
PS Catulene	70.8%	CS Savane	79.2%	13	CS Chueza	14	HR Nhamatanda	CS Mangunde	84.7%	CS Maringuê	83.3%	12	CS Savane	11	CS Mazamba
CS Maringuê	70.8%	CS Chingussura	76.4%	14	CS Chingussura	15	CS Caia	CS Ponta Gêa	80.6%	HR Marromeu	83.3%	13	CS Inhaminga	11	CS Savane
CS Ponta Gêa	66.7%	CS Machanga	68.1%	15	HR Nhamatanda	15	CS Inhaminga	CS Chemba	80.6%	CS Muanza	83.3%	14	CS Machanga	11	CS Gorongosa
CS Chingussura	66.7%	CS Dondo	66.7%	16	CS Muanza	15	CS Dondo	CS Chingussura	77.8%	CS Caia	77.8%	15	PS Catulene	11	CS Canxixe
CS Tica	65.3%	CS Maringuê	66.7%	17	PS Catulene	15	CS Machanga	CS Canxixe	77.8%	CS Savane	76.4%	16	CS Murema	12	CS Maringuê
HR Nhamatanda	62.5%	HR Marromeu	66.7%	17	CS Gorongosa	15	HR Marromeu	CS Gorongosa	72.2%	CS Gorongosa	76.4%	17	CS Gorongosa	13	HR Muxunguê
CS Machanga	50.0%	HR Nhamatanda	65.3%	17	CS Machanga	15	CS Muanza	PS Catulene	63.9%	CS Canxixe	70.8%	17	CS Canxixe	14	CS Muanza

DIRECÇÃO PROVINCIAL
DE SAÚDE DE SOFALA
