# Supplementary material for: Strengthening integrated primary health care in Sofala, Mozambique
Source: BMC Health Serv Res. 2013 May 31;13(Suppl 2):S4. doi: 10.1186/1472-6963-13-S2-S4 (PMC3668215; doi:10.1186/1472-6963-13-S2-S4)
Supplement: Additional file 2 [file 1472-6963-13-S2-S4-S2.pdf]

DIRECÇÃO PROVINCIAL DE SAÚDE DE SOFALA

MOÇAMBIQUE

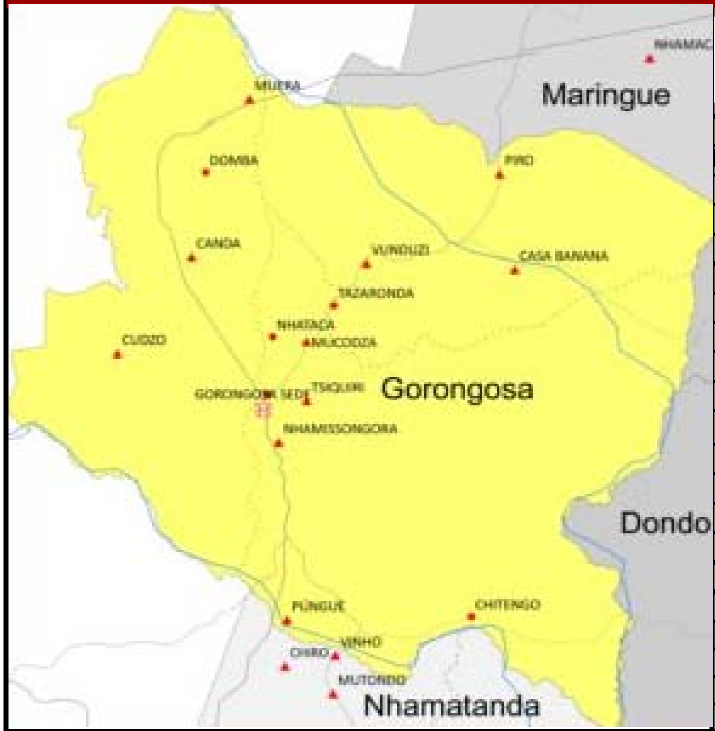

RETRO INFORMAÇÃO GORONGOSA 2010/2011

POPULAÇAO - 2011	
População - Distr	138542
Grávidas Previstas	6288
Partos Previstos	6234
Crianças 0-11 meses	5031

REDE SANITARIA	
Tipo	Nº
Hospital Central	0
Hospital Rural	0
C.S. Urbano A	0
C.S. Urbano B	0
C.S Urbano C	0
C.S Rural I	1
C.S. Rural II	10
Posto de Saúde:	3
**Total**	**14**

R-HUMANOS (DISTRITO)	
Médicos	1
Técnico Superiores	1
Téc. Medicina Geral	9
Tec. Medicina Preventiva	3
Enfermeiras SMI - Médio	3
Enfermagem - Médios	3
Outros - Médios	5
Administrativos - Médios	0
Enfermeiras SMI - Básicas	10
Enfermagem - Básicas	11
Outros - Básicos	12
Administrativos - Básicos	1
Agentes de Serviço	33
Outros	5
**Total**	**97**

RECURSOS MATERIAIS (DISTRITO)- DEZ/2011

	Funcionais	Avariadas	Total
Viaturas	3	1	4
Motorizadas	3	12	15
Geleiras - PAV	7	2	11

1a CPN; PARTOS INST.; E P. FAMILIAR

UNIDADES SANITÁRIA	1☐ CPN		P. INST		P. FAM	
	2010	2011	2010	2011	2010	2011
Canda	1161	908	435	399	733	192
Casa Banana	202	242	17	37	7	31
Cudzo	218	354	18	102	36	26
Gorongosa	2961	2970	1889	1820	744	1293
Mucodza	1046	976	401	334	237	175
Muera	5	133	1	8	4	65
Nhamissongora	272	220	20	30	79	46
Piro	425	454	161	219	160	123
Púngué	651	545	213	225	64	68
Tsiquir	381	325	99	31	120	91
Vunduzi	650	521	240	236	100	65
**Total Distrital**	**7972**	**7648**	**3494**	**3441**	**2284**	**2175**

Planeamento Familiar :Novas utentes

Vermelho: Redução de produtividade >10%

COBERTURA: CPN; PARTOS INST. E P. FAMILIAR- 2011

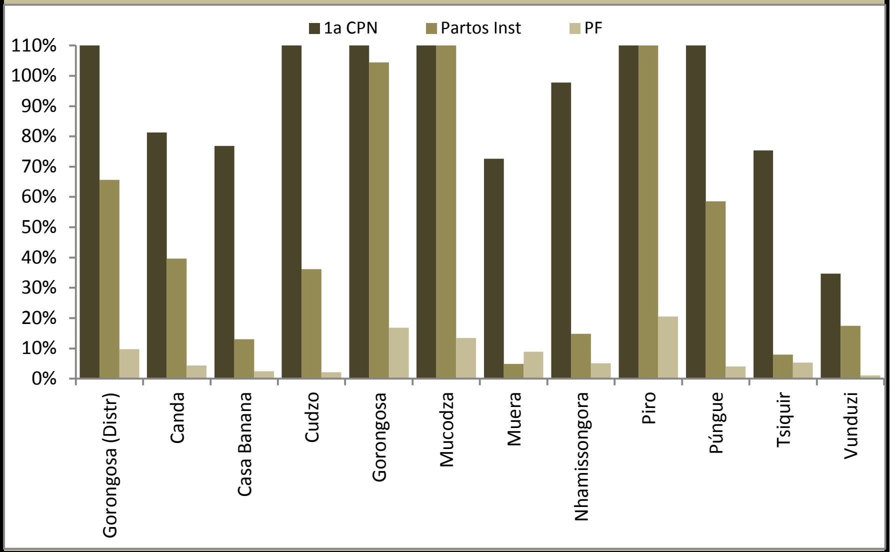

CONSULTA SMI - (PTV) - 2011

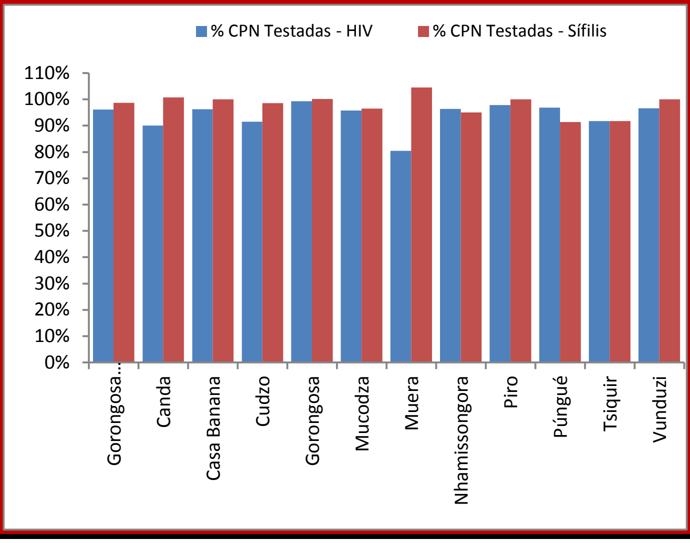

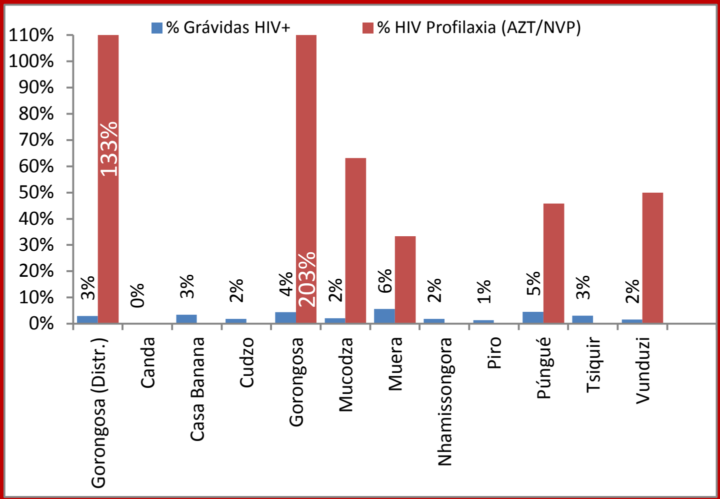

Perca de oportunidades da profilaxia em 7 Unidades Sanitárias

RETRO - INFORMAÇÃO 2010/2011 - Gorongosa

COBERTURA - PAV - 2011

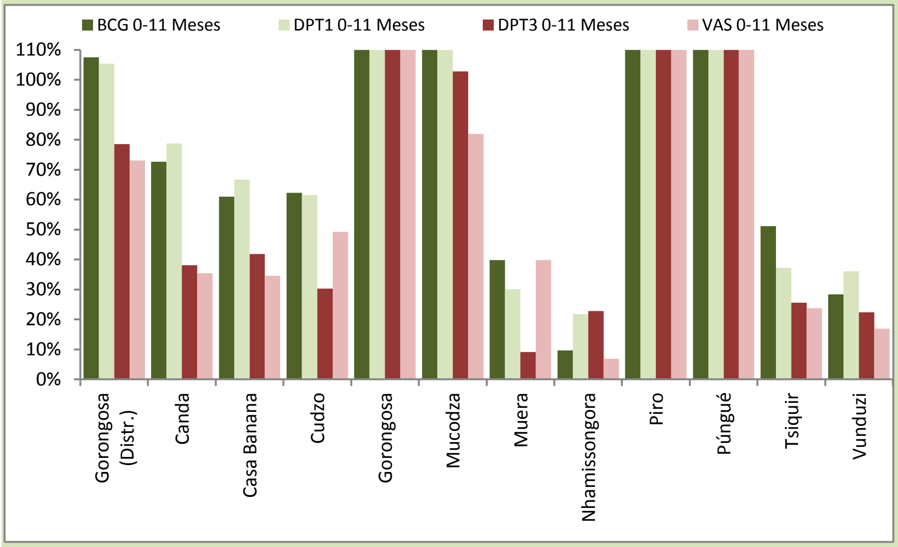

Todas US com cobertura abaixo de 100% menos o CS Gorongosa

NUTRIÇAO (Distr.)

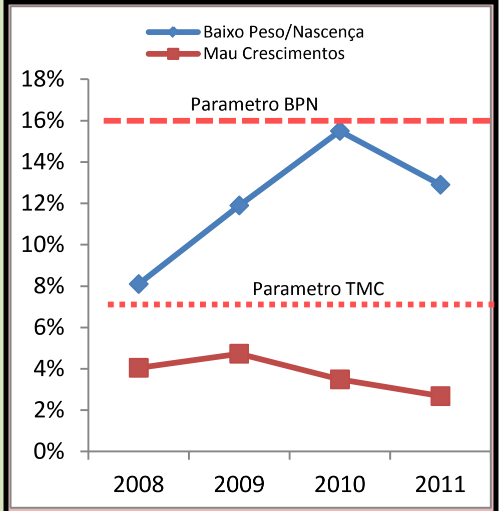

Todos indicadores abaixo do limiar em quatro anos consecutivos

CONSULTAS EXTERNAS REALIZADAS

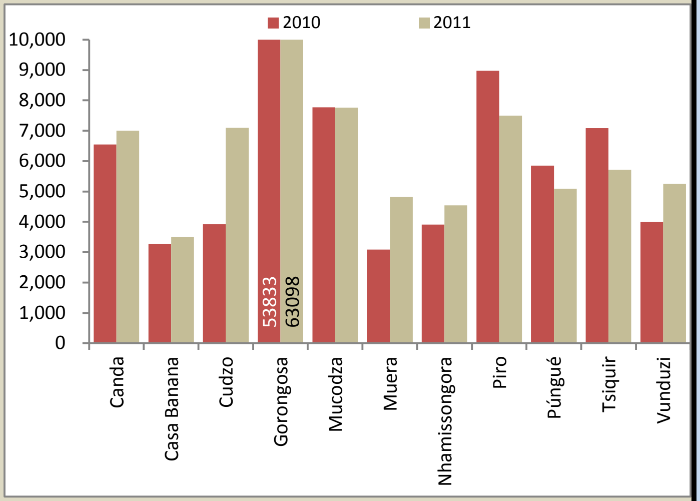

Aumento de consultas >10% nas seguintes US. Gorondosa Sede, Cudzo, Vunduzi, Muera e Nhamissomgora

CASOS DA DIARREIA NOTIFICADOS-BES

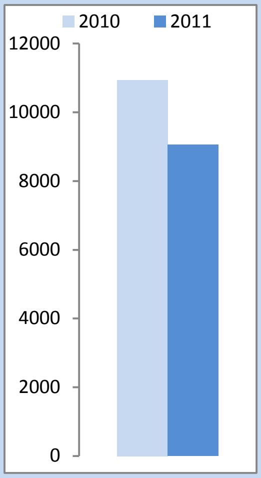

Reduçao da diarreia em 20%

CASOS DE MALÁRIA CONFIRMADOS E NOTIFICADOS 2011-BES/LAB

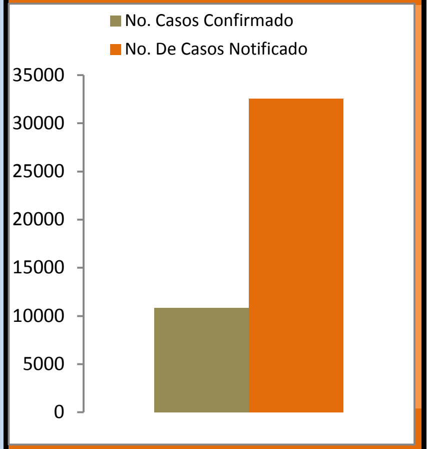

Diferença de 21740 entre casos notificados e confirmados

Percentagem de Disponibilidade e Concordância Alta

Indicadores	Gorongosa 2009	Gorongosa 2010	Mucodza 2009	Mucodza 2010
**Partos Institucionais**				
Disponibiliadde	80.0%	75.0%	98.3%	93.3%
Alta Concordância	90.9%	100%	97.2%	100%
**Consultas Externas**				
Disponibiliadde	73.3%	70.0%	76.7%	90.0%
Alta Concordância	12.5%	-	82.4%	97.1%
**1a Consulta Pré-Natal**				
Disponibiliadde	77.8%	87.5%	95.8%	95.8%
Alta Concordância	-	86.5%	88.6%	82.2%
**3a Dose DPT HepB-HIB**				
Disponibiliadde	72.2%	76.4%	94.4%	95.8%
Alta Concordância	-	74.1%	72.7%	68.6%

Disponibilidade de Material Essencial

NB:(n=3)	Disponibilidade	Ruptura últimos 3 meses
Cotrimoxazole	3 (100%)	0 (0.0%)
Coartem Criança	2 (100%)	1 (50.0%)
Coartem Adulto	3 (100%)	1 (33.3%)
Sal Ferroso	3 (100%)	2 (66.7%)
Plumpy Nut	3 (100%)	0 (0.0%)
TARV (1a Linha)	2 (100%)″	0 (0.0%)
DPT	2 (66.7%)	1 (33.3%)
Preservativos	2 (66.7%)	1 (33.3%)
Luvas de Exame	3 (100%)	0 (0.0%)
Geleiras	1 (50.0%)	1 (50.0%)
Teste de Sífilis	3 (100%)	0 (0.0%)
TR Malária	3 (100%)	1 (33.3%)
Determine	0 (0.0%)	3 (100%)

TAXA DE DESPISTE E CURA

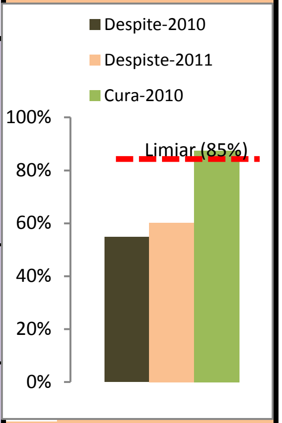

Despiste abaixo do limiar em dois anos consecutivos

Fonte: Módulo Básico; Programas, Estudos CIOB

REPÚBLICA DE MOÇAMBIQUE
GOVERNO DA PROVÍNCA DE SOFALA
DIRECÇÃO PROVINCIAL DE SAÚDE

HEALTH ALLIANCE INTERNATIONAL
